# Evaluation of the role of combining inter-arm systolic pressure difference and derivatives of pulse volume recording in detecting subclavian artery stenosis

**DOI:** 10.3389/fcvm.2022.962610

**Published:** 2022-09-15

**Authors:** Xuanqi An, Hui Dong, Yu Deng, Yang Chen, Yubao Zou, Weiguo Zhang, Xiongjing Jiang

**Affiliations:** ^1^Department of Cardiology, Fuwai Hospital, National Center for Cardiovascular Disease, Chinese Academy of Medical Sciences, Peking Union Medical College, Beijing, China; ^2^Las Colinas Institutes, Irving, TX, United States

**Keywords:** atherosclerosis, subclavian artery stenosis, inter-arm systolic pressure difference, pulse upstroke time, pulse upstroke time per cardiac cycle

## Abstract

**Background:**

Subclavian artery stenosis (SAS) is a peripheral arterial disease of asymptomatic appearance and disastrous consequences. The traditional screening method remains unsatisfactory.

**Objective:**

The study aimed to assess the diagnostic performances of inter-arm systolic pressure difference (IASBPD), derivatives of pulse volume recording (PVR), and their combination in detecting subclavian artery stenosis.

**Materials and methods:**

The present study was a retrospective analysis of clinical data from inpatients suspected of supra-arch artery stenosis in Fuwai hospital during 1 year, who underwent selective arterial angiographies. We obtained simultaneous blood pressure measurements on four limbs and pulse waves for calculating IASBPD and PVR derivatives prior to the angiographies. We utilized the receiver operating characteristic curve (ROC) to calculate the optimal cut-off value of IASBPD, upstroke time (UT), and upstroke time per cardiac cycle (UTCC) for detecting SAS. Moreover, we compared the sensitivity and specificity of IASBPD, UT, UTCC, and their combinations for diagnosing SAS (Clinical trial number: NCT03521739).

**Results:**

We consecutively enrolled 320 eligible patients. Based on SAS’s definition of stenosis above 50%, the area under the curve of IASBPD, UT, and UTCC were 0.84, 0.76, and 0.80 (*P* < 0.001). And their corresponding cut-off points were 9 mmHg, 202 milliseconds, and 23.2%. The sensitivity and specificity of IASBPD ≥ 9 mmHg were 57.0 and 94.1%. UT ≥ 202 ms and UTCC ≥ 23.2% yielded similar sensitivity (72.6 vs. 72.6%, *P* > 0.05), but UTCC had higher specificity (81.1 vs. 72.4%, *P* < 0.05). The sensitivity of the combination of IASBPD and UT (85.2%) or UTCC (78.5%) was significantly higher than IASBPD alone (57%, *P* < 0.05). The specificity of either combination decreased to 67.6 and 76.8% (*P* < 0.05).

**Conclusions:**

This present study showed that the combinations of IASBPD and PVR-derived parameters promoted diagnostic sensitivity and preserved adequate specificity than those alone for detecting SAS.

## Introduction

Subclavian artery stenosis (SAS) is a peripheral arterial disease mainly caused by atherosclerosis. Its prevalence is around 1.5% in the general population and has significantly increased as the aging society exacerbates worldwide. (1–3). SAS can cause upper limb ischemia and further reduce cerebral blood flow due to the subclavian artery steal syndrome (SSS). It also triggers ischemic posterior circulation stroke or even coronary SSS in the patient with coronary artery bypass grafting surgery (CABG), where the left internal mammary artery (LIMA) is grafted (4). The early detection of SAS remains the cornerstone of the systematic risk evaluation, treatment, and follow-up of the atherosclerotic burden from a cardiovascular perspective (1–6).

The inter-arm systolic pressure difference (IASBPD) has been widely used in detecting SAS due to its non-invasive nature and convenience. However, the relatively small epidemiological data on IASBPD has yet to establish an optimal cut-off value (1–3, 5, 7, 8). Moreover, it might also produce false-negative results in patients with bilateral SAS or mild SAS. Therefore, in recent years, simultaneous measurement of four limbs’ blood pressure, pulse volume recordings, and its derivatives, such as pulse wave upstroke time (UT) and upstroke time per cardiac cycle in percentage (UTCC), have been increasingly adopted to evaluate vascular pathophysiology comprehensively (9–14). Not affected by the blood pressure of the contralateral side, UT generally increases when the upstream artery narrows. Moreover, studies show that using UT has enhanced the detection rate of lower extremity arterial stenosis (10–12). Furthermore, the use of UTCC has improved the diagnostic accuracy of UT by minimizing the impact of heart rate on UT (11).

The present study aims to assess the diagnostic value of IASBPD, UT, and UTCC, emphasizing their sensitivity and specificity alone or when combined in detecting SAS.

## Materials and methods

### Study population

The Ethics Committee in our institution has approved the study. Our study is a retrospective analysis of the population from the cohort of “Chinese Registered Study on Synchronizing Extremity Blood Pressure and Pulse Wave Velocity and Cardiovascular Outcomes, ASORPWICE” (Registration Number: NCT03521739). The entire protocol can be accessed online at ClinicalTrials.gov. We consecutively included the patients suspected of superior arch artery stenosis and later confirmed by angiography in the Fuwai hospital from October 2017 to October 2018. Apart from signing standard informed consent regarding the disease and the interventional procedure, all patients agreed to participate in the study after they were fully informed about the research plan. All participants had records of baseline characteristics, including age, sex, ID number, contacts information, relevant risk factors, smoking habits, left ventricular ejection fraction measured by echocardiography, medications, and lab test results such as hemoglobin levels, cardiac enzymes, and liver and kidney functions both before and after the intervention. We also collected simultaneous blood pressure measurements in the four limbs, the pulse waves, and the supra-arch angiographies results. Those who met the following criteria were excluded: (1) Moderate to severe aortic valve stenosis or left ventricular outflow tract obstruction. (2) Diseases that caused severe arrhythmia and significant blood pressure fluctuations during the measurements, such as atrial fibrillation. (3) There are missing limbs, limb malformation, or limb pain at the blood pressure measurement site. (4) Non-atherosclerotic arterial disease, such as Takayasu arteritis. (5) Missing data of IABPSD, PVR derivatives, or angiographies.

### Limb blood pressure measurement and pulse volume recording

In a quiet clinical office with a temperature of 22–25°C, we collected the patient’s general information (e.g., age, gender, height, and weight). The subjects then took a relaxed supine position, with upper limbs on both sides of the body, and rested for 5 min. We then attached ECG and heart sound sensors to the patients. As previously described in other literature, We adopted the extremity blood pressure meter (VP-1000 plus, Omron, Japan) to record the measurements (9, 13, 14). First, the four blood pressure cuffs were placed on the upper arms and ankles. The lower edge of the upper arm cuff was 2 cm above the elbow socket, with the air cuff mark set above the brachial artery. The tightness of each cuff should allow placing two fingers in the space under the cuff. Next, we inflated all the cuffs of the limbs simultaneously, recorded the pulse wave of the limbs continuously, and started the measurements after the sensor output waveform stabilized.

**FIGURE 1:**
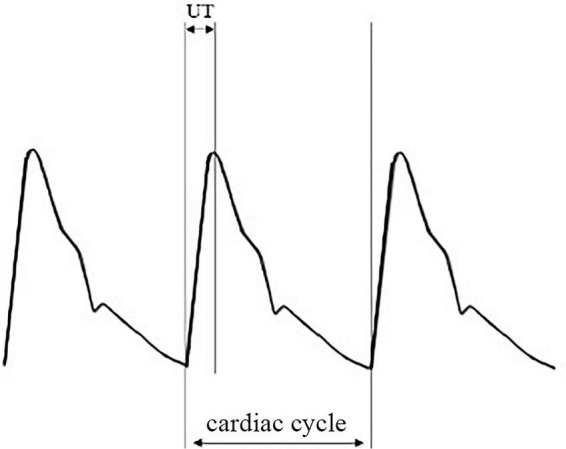
An illustration of pulse volume recording (PVR) and its derivative parameters. PVR, pulse volume recording or pulse volume chart; UT, pulse wave upstroke time or pulse wave ascending time, which is the time required from the beginning to the peak of an arterial pulse wave in milliseconds (ms); UTCC, upstroke time per cardiac cycle time in percentage.

Nurses with professional training performed these procedures. We defined IASBPD as the absolute difference in systolic pressure of the bilateral brachial arteries. We measured UT and UTCC based on brachial arteries. And we selected the highest value among the limbs to report. Figure 1 showed a typical pulse volume recording (PVR), where UT was the pulse wave upstroke (ascending) time, and UTCC was the ratio of UT to the length of the cardiac cycle (Figure 1).

### Subclavian arteriography and calculation of the stenosis percentage

According to the common standard, we performed the subclavian arteriography three to five days after the IABPSD and PVR derivative measurements (6). In brief, after local anesthesia, we inserted the catheter into the proximal end of the brachiocephalic trunk at 40–50° angles through the femoral artery approach to obtain the right subclavian arteriography image. Next, the catheter was inserted into the proximal end of the left subclavian artery to get the left subclavian artery angiography image. Finally, we adjusted the X-ray projection until we achieved an angiographic image of good quality. We diagnosed SAS when the stenosis of subclavian artery diameter was equal to or greater than 50% from angiography. The experienced interventional cardiologists in our center conducted the procedures with an angiographic X-ray system–GE Innova IGS 520 (GE Company, United States).

**FIGURE 2:**
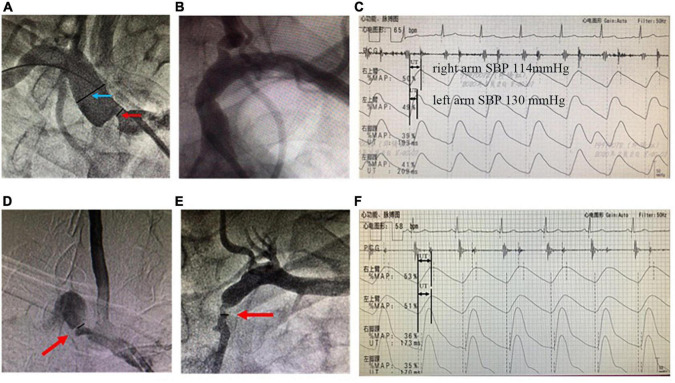
Angiographies of SAS and recordings of simultaneous extremity blood pressure and PVR. The upper panels are from a representative unilateral SAS case. **(A)** Arteriography showed the stenotic severity of the proximal lumen of the right subclavian artery. The degree of stenosis was 71.4% (the diameter of the narrowest lumen in the red arrow/the reference diameter of the distal lumen in the blue arrow; **(B)** the lumen of the left subclavian artery was normal; **(C)** The simultaneous extremity blood pressure measurement showed inter-arm systolic blood pressure difference = 16 mmHg (right arm systolic pressure 114 mmHg, left arm systolic pressure 130 mmHg). And PVR showed that the peak of the right arm was delayed significantly more than that of the left arm. The UT and UTCC were 260 ms and 28.2%, respectively. While the left arm UT and UTCC were 184 ms and 19.9%, respectively. The lower panels are from a bilateral SAS case. **(D)** Arteriography shows right SAS of 76.4%; **(E)** left SAS of 69.1%; **(F)** the UT of the left and right arms are 309 and 322 ms, and the UTCC is 29.9 and 31.1%, respectively. SAS, subclavian artery stenosis; IASBPD, inter-arm systolic blood pressure difference; please see previous figures or tables for other abbreviations.

We used electronic vernier calipers to measure the diameters of the narrowest lumen of the stenosis and the normal lumen at the distal to the stenosis. We adopted the NACSET standard to calculate the degree of stenosis: the degree of stenosis = (1-the narrowest lumen diameter/distal normal lumen diameter) × 100% (Figure 2).

### Statistical methods

We performed all statistical analyses using SPSS 25.0 software. Continuous variables were expressed by mean ± standard deviation (SD) and compared by the student’s *t*-test. Categorical variables were represented by frequency and percentage and compared by the χ2 test or Fisher’s exact probability method. The area under the curve (AUC) was calculated using the receiver operating characteristic curve (ROC). We used the Youden index as the cut-off point for the diagnosis of SAS ≥ 50% to calculate the sensitivity, specificity, positive predictive value, negative predictive value, and the diagnostic accuracy of IASBPD, UT, UTCC, and the combination of IASBPD and UT or UTCC in detecting SAS. We considered a *P*-value less than 0.05 to be statistically significant.

### Patient and public involvement statement

Patients or the public WERE NOT involved in our research design, conduct, reporting, or dissemination plans.

## Results

### Patient baseline characteristics, angiography results, blood pressure of the four limbs, and pulse volumetric measurements

We retrospectively analyzed 320 patients (640 subclavian arteries) with 248 males (77.5%) in the study. The enrolled participants had a mean age of 64.3 ± 8.5 years (ranging from 39 to 84 years old). As depicted in Table 1, Most of the SAS patients had a history of hypertension (78.8%), hyperlipidemia (78.4%), active smoking (62.2%), and concomitant coronary heart disease (62.2%). We then divided the patients into different groups based on the severity of SAS. One hundred eighty-five patients had subclavian artery atherosclerosis of less than 50%, while 135 patients out of 320 (42.2%) with 169 subclavian arteries had SAS. Among them, 34 patients had bilateral stenosis, and 101 cases had unilateral stenosis, with 65 left and 36 right side stenosis. Furthermore, 99 out of 135 had SAS of 70 to 99%, and 17 patients had utterly occluded subclavian arteries.

**TABLE 1:** General characteristics of 320 patients with supra-arch angiograph.

Baseline characteristics	
Age (years)	64.3 ± 8.5
Male	248 (77.5%)
Body mass index	24.6 ± 2.78
Cases with hypertension	252 (78.8%)
Cases with hyperlipidemia	251 (78.4%)
Cases with diabetes	122 (38.1%)
Cases with smoking	199 (62.2%)
Cases with coronary heart disease	199 (62.2%)
Cases with stroke	53 (16.6%)
Cases with left ventricular ejection fraction <45%	43 (13.4%)
Cases with serum creatinine >144 μmol/L	11 (3.4%)

Values are shown as absolute numbers (percentage) and mean ± standard deviation.

We measured IASBPD, UT, and UTCC in all 320 SAS patients. No adverse events occur during the measurements. The measured values of IASBPD, UT, and UTCC corresponding to different degrees of SAS patients and their comparison are shown in Table 2. In brief, compared with individuals with mild SAS, severe SAS patients had more pronounced IASBPD, longer UT, and higher UTCC. The differences in UT and UTCC across groups were all considered statistically significant. The differences in IASBPD between SAS of 70 to 99.9% or 100% and SAS of 0 to 49.9% were also statistically significant.

**TABLE 2:** The relationship between stenosis severity and relevant SAS parameters.

Test results	SAA of 0–49.9%	SAS of 50–69.9%	SAS of 70–99.9%	SAS of 100%	*P*-value
Number of patients	185 (57.8)	19 (5.9)	99 (30.9)	17 (5.3)	0.000
Number of upper limbs	471 (73.6)	37 (5.8)	115 (18.0)	17 (2.7)	0.000
UT (ms)	172.1 ± 37.9	188.5 ± 39.3*	215.0 ± 39.7***	240.0 ± 38.1***	0.003
UTCC (%)	19.4 ± 3.6	21.2 ± 4.0*	25.2 ± 4.8***	30.0 ± 5.4***	0.000
IASBPD (mmHg)	3.9 ± 3.4	6.2 ± 3.3	15.6 ± 13.4***	37.4 ± 19.7***	0.000

Values are shown as absolute numbers (percentage) and mean ± standard deviation. Compared with SA of 0–49.9%, *P < 0.05, ***P < 0.001. The *P*-value for each row indicates the *P*-value across four groups. SAA, subclavian artery atherosclerosis; SAS, subclavian artery stenosis; UT, pulse wave upstroke time; UTCC, pulse wave upstroke time/cardiac cycle time in percentage; IASBPD, inter-arm systolic pressure difference; please see previous figures or tables for other abbreviations.

### The sensitivity and specificity of inter-arm systolic pressure difference, upstroke time, upstroke time per cardiac cycle, and their combinations in detecting inter-arm systolic pressure difference

**FIGURE 3:**
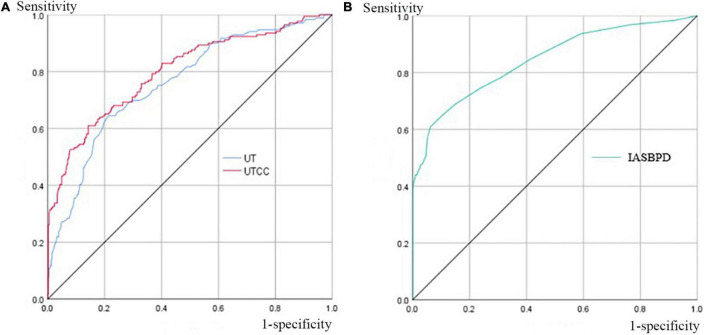
Area under the curves (AUCs) of UT, UTCC, and IASBPD when SAS ≥ 50%. Panel **(A)** the UT and UTCC curves when SAS is equal to or greater than 50%, and their corresponding AUC is 0.76 (95% CI: 0.72–0.80) and 0.80 (95% CI: 0.76–0.85) for UT and UTCC, respectively. Panel **(B)** the IASBPD curve, when SAS is equal to or greater than 50%, corresponds to 0.84 (95% CI: 0.80–0.89). AUC, the area under the curve; CI, confidence interval; please see previous figures or tables for other abbreviations.

When we set the cut-off point of IASBPD as 9 mmHg, it achieved the maximum diagnostic power in detecting SAS with an AUC of 0.84 (95% CI: 0.80–0.89, *P* < 0.001). Its sensitivity was 57.0%, and its specificity was 94.1% (Figure 3 and Table 3). The sensitivity of diagnosing unilateral SAS was 66.3%, while diagnosing bilateral SAS patients was only 29.4% (Table 4). When IASBPD was set at the cut-off point of 10 mmHg as the recommended diagnostic cut-off point internationally (15), its sensitivity decreased significantly while specificity increased slightly without significance (Table 3).

**TABLE 3:** Sensitivity and specificity of IASBPD, UT, and UTCC alone and their combinations in screening SA.

	Sensitivity	Specificity	Positive	Negative	Accuracy
IASBPD ≥ 9 mmHg	57.0	94.1	87.5	75.0	78.4
IASBPD ≥ 10 mmHg	52.6*	94.6	87.7	73.2	76.9
UT ≥ 202 ms	72.6**	72.4***	65.8	78.4	72.5
UTCC ≥ 23.2%	72.6**	81.1**	73.7	80.2	77.5
IASBPD and UT	85.2***	67.6***	65.7	86.2	75.0
IASBPD and UTCC	78.5***	76.8***^#^	71.1	83.0	77.5

Values are shown as percentages. *P < 0.05, **P < 0.01, ***P < 0.001 when compared with IASBPD ≥ 9 mmHg. *P* < 0.05 when compared with UT ≥ 202 ms. ^#^*P* < 0.05 when compared with IASBPD and UT. Please see previous figures or tables for abbreviations.

**TABLE 4:** The sensitivity of IASBPD alone and its combination with UT or UTCC in screening unilateral and bilateral SAS.

SAS	Unilateral	Bilateral	All
Number	101	34	135
IASBPD ≥ 9 mmHg	67 (66.3)	10 (29.4)	77 (57.0)
IASBPD and UT	91 (90.1)***	24 (70.6)**	115 (85.2)***
IASBPD and UTCC	86 (85.1)**^#^	20 (58.8)*^#^	106 (78.5)***^#^

Values are shown as absolute numbers (percentage). *P < 0.05, **P < 0.01, ***P < 0.001 when compared with IASBPD ≥ 9 mmHg. *P* < 0.05 when compared with UT ≥ 202 ms. ^#^*P* < 0.05 when compared with IASBPD and UT. Please see previous figures or tables for abbreviations.

When the UT cut-off point was set at 202 milliseconds (ms) with AUC 0.76 (95% CI: 0.72–0.80, *P* < 0.001), its sensitivity was 72.6%, and specificity was 72.4% (Figure 3 and Table 3). If we set the UTCC cut-off point as 23.2%, its sensitivity was 72.6%, and the specificity was 81.1% (Figure 3 and Table 3). The difference between UT and UTCC did not reveal any statistical significance in the aspect of AUC and sensitivity in detecting SAS. However, UTCC showed greater specificity (*P* < 0.05). In addition, compared with IASBPD, both UT and UTCC showed higher sensitivity (*P* < 0.01) and lower specificity (*P* < 0.01) (Table 3).

The sensitivity of combining IASBPD and UT was higher than that of IASBPD and UTCC without statistical significance (85.2 vs. 78.5%, *P* > 0.05). In contrast, the specificity of the combination of IASBPD and UT was significantly lower than that of IASBPD and UTCC (67.6 vs. 76.8%, *P* < 0.05). The combination of IASBPD and UT or UTCC significantly improved sensitivity in detecting SAS than IASBPD, UT, or UTCC alone. However, the combinations reduced the specificity (*P* < 0.001, Table 3). As Figure 2 illustrates, The second patient developed severe stenosis of the bilateral subclavian arteries. His IABPSD was 5 mmHg within the normal range, while the UT and UTCC were significantly higher bilaterally.

### The sensitivity of inter-arm systolic pressure difference with upstroke time or with upstroke time per cardiac cycle in detecting unilateral and bilateral subclavian artery stenosis

Compared with IASBPD alone, the combinations of IASBPD and UT or UTCC significantly enhanced the sensitivity of detecting both unilateral (90.1 vs. 66.3%, *p* < 0.001; 85.1 vs. 66.3%, *p* < 0.001) and bilateral SAS (70.6 vs. 29.4, *p* < 0.001; 58.8 vs. 29.4%, *p* < 0.05) (Table 4).

## Discussion

Having enrolled the largest cohort of 320 SAS patients, The present study evaluated the sensitivity and specificity of IASBPD, PVR derivatives, and their combinations in detecting SAS. For IASBPD, Our study achieved a larger AUC and a greater sensitivity when the cut-off point of IASBPD was 9 mmHg instead of 10 mmHg. The better cut-off points for UT and UTCC on upper limbs were 202 ms and 23.2%. In addition, Our results demonstrated that UT and UTCC were valuable tools for detecting upper extremity arterial stenosis. Furthermore, the combination of IASBPD and UT or UTCC significantly improved the diagnostic sensitivity of SAS than IASBPD, UT, or UTCC alone, especially in the case of bilateral SAS.

Because asymptomatic SAS cases are often overlooked in clinical practice, convenient and effective screening methods are direly needed. In 2001, English et al. (7) analyzed the left subclavian angiography results of 492 individuals who primarily received coronary angiography. For 17 cases with SAS out of 492 participants, the study found that if the cut-off point of IASBPD was 10 mmHg, its sensitivity was 65%, and its specificity was 85% for detecting SAS above 60%. In 2002, having set the cut-off point of IABPSD as 15 mmHg, Osborn et al. (8) identified four patients with SAS above 50% out of 59 participants who were to receive cardiac bypass graft surgery. Later, Clark et al. recommended that the appropriate cut-off point of IASBPD for screening SAS was 10 mmHg (15). However, previous studies inherited several limitations: (1) Sample sizes were often small. (2) The blood pressure on the upper arms was not measured simultaneously. (3) The studies did not utilize the AUC to calculate the optimal cut-off point of IASBPD. The present study overcame these shortcomings and solidified our results. We included 320 SAS patients and simultaneously measured the blood pressure in the upper arms. Furthermore, we have utilized AUC for cut-off value calculations. For our results, we testified that the optimal cut-off value for IASBPD to diagnose SAS above 50% is 9 mmHg rather than 10 mmHg, which is currently recommended in the literature. Our data supported that the cut-off value of 9 mmHg improved IASBPD’s diagnostic sensitivity without undermining its specificity.

Another concern of IASBPD in diagnosing SAS is the false-negative result, especially when the patient had bilateral SAS of a similar degree (2, 4). UT and UTCC, the upper arm PVR derivatives, reflect the change of the pressure waveform downstream of the stenosis, which is mainly affected by the degree of stenosis upstream of the ipsilateral brachial artery. Therefore, their immunity to the influence of the stenosis on the contralateral upper extremity artery serves as a critical advantage over IASBPD in detecting bilateral SAS. Our study studied the use of PVR derivatives and confirmed that the sensitivity of UT or UTCC in diagnosing SAS was significantly higher than that of IASBPD. The results also depicted that combining IASBPD and UT or UTCC significantly improved the sensitivity of diagnosing bilateral SAS than IASBPD, UT, or UTCC alone. However, it is worth noting that the specificity of the combinations decreased slightly within the acceptable range, especially when IASBPD and UT were combined.

Instead of IABPSD, UT, or UTCC alone, The study results supported combining IABPSD and UT or UTCC in detecting SAS. As listed above, the sensitivity of the combination of IASBPD and UT or UTCC raised significantly more than those alone. At the same time, the specificity of the combinations decreased slightly to 76.8% (IABPSD and UTCC) and 67.6% (IABPSD and UT). We believe the specificity of the combination is adequate and acceptable for clinical practice.

Our study possesses several limitations. First, the study is single-centered and may induce selection bias. However, our study aims to evaluate the diagnostic power of IABPSD, UT, and UTCC in SAS patients. Moreover, our study has enrolled patients with confirmed SAS. So selection bias is limited. Still, the issue of external validity resides due to the nature of the single-centered design. In addition, We excluded individuals with atrial fibrillation because the condition would interfere with the PMR for our exclusion criteria. We also reject participants with non-atherosclerotic causes such as Takayasu arteritis. The exclusions will also limit the external validity. Another issue of concern is observational bias. Pitfalls may lie in diagnosing SAS by relying solely on PVR-derived parameters. First, UT measurement might produce false-positive results in patients with severe aortic valve or left ventricular outflow tract obstruction (16). Careful auscultation and echocardiography are needed to differentiate the condition. We have excluded patients with severe aortic valve or left ventricular outflow tract obstruction during enrollment in the present study by reviewing the echocardiography results. Second, in patients with severe aortic stiffness, the amplified reflecting pressure waves could superimpose on the initial pressure wave, prolonging the UT and leading to false-positive results (17). Moreover, the measuring equipment cannot identify the false peak in the pulse waveform, so manual measurements by medical professionals are required. All PVR measurement results have been manually checked for our study to minimize observational bias. Third, the measurement of UT is prone to the influence of heart rate. Bradycardia can prolong UT, while tachycardia shortens the UT. Defined as the percentage of UT in a cardiac cycle, UTCC is an ideal substitute for UT in our study (16). Atrial fibrillation could also interfere with the PVR readings. Nevertheless, we have excluded patients with atrial fibrillation at the beginning. Last but not least, the simultaneous limb blood pressure and pulse wave are hemodynamic measurements. While they can reflect whether there is severe stenosis in the upstream arteries, they cannot infer the specific location, anatomical features, and nature of the stenosis (4). In our study, all the participants had lab test results and angiographies records to determine SAS’s specific location and nature.

In summary, our retrospective study supported that combining IASBPD and PVR-derived parameters, including UT and UTCC, provided superior sensitivity and acceptable specificity in detecting SAS. Therefore this method could be a convenient, non-invasive, and cost-effective alternative in detecting individuals with SAS in routine cardiovascular practice.

## Data availability statement

The raw data supporting the conclusions of this article will be made available by the authors, without undue reservation.

## Ethics statement

The studies involving human participants were reviewed and approved by the Ethics Committee of Fuwai Hospital. The patients/participants provided their written informed consent to participate in this study.

## Author contributions

XA and HD drafted the manuscript. HD, YD, and YC conducted the study. YZ and WZ supervised the commencement of the study. XJ was the director of the department of vascular disease, was in charge of the investigation, and he helps edited the manuscript. All authors contributed to the article and approved the submitted version.
